# Exploring the Effectiveness of Sustainability Measurement: Which ESG Metrics Will Survive COVID-19?

**DOI:** 10.1007/s10551-022-05183-1

**Published:** 2022-08-04

**Authors:** Jill Atkins, Federica Doni, Andrea Gasperini, Sonia Artuso, Ilaria La Torre, Lorena Sorrentino

**Affiliations:** 1 Cardiff Business School, Cardiff University, Cardiff, UK; 2 Department of Business & Law, University of Milano-Bicocca, Milan, Italy; 3 AIAF, Milan, Italy; 4 CSR Europe, Brussels, Belgium

**Keywords:** Pandemic crisis, ESG disclosure, Metric, Reactivity, Institutionalism, Resilience

## Abstract

This paper aims to investigate the current state of play on Environmental Social and Governance (ESG) integration and check the validity of the current metrics system by assessing if it will survive the COVID-19 crisis. By adopting a qualitative research approach through semi-structured anonymous interviews with 14 senior managers of six European listed companies we use a framework by assessing the mechanisms of reactivity on the effectiveness of ESG measures in times of COVID-19. By interpreting the practitioners’ points of view through the lens of the sociological framework by Espeland and Sauder (Am J Sociol 113:1–40, 2007) our findings show different mechanisms of reactivity by companies on the effectiveness of ESG measures in times of COVID-19, i.e., active and passive conformity and active resistance. We also identified the main Corporate Social Responsibility (CSR) institutional factors that affect managers’ reactivity. An extensive re-formulation of the ESG metrics is required in the light of times of crisis, given that accountability and transparency are strongly linked to quantitative measures which can play a critical role in the financial system and investors’ engagement. Particularly, the strict distinction between “E”, “S” and “G” issues should be abandoned claiming a different holistic re-design of sustainability measures by considering the increasing relevance of the Social dimension in time of COVID-19. This study provides a valuable contribution to the existing literature on the measurement of sustainability within the link of accountability and crisis by highlighting new corporate needs to re-design the ESG metrics system.

## Introduction

Climate change and the necessary energy transition, as described in the *European Green Deal* (EU, 2020a) of the European Union (EU), are clear and well-known issues and can no longer be considered a secondary problem even in the situation of the health and economic crisis of Covid-19. The economic recovery must start from the real economy and from those sectors that present more just and inclusive activities with low environmental impact, resilient to climate change.

Among several studies aimed at highlighting the link between climate and health (Watts et al., 2019,1; IPCC, 2019,2), the World Economic Forum (WEF, 2021) in the recent Global Risks Report 2021 also highlights that climate change is the greatest threat to global health in the twenty-first century and exacerbates the incidence of infectious diseases.^3^

The current crisis calls for a systemic approach that pays attention to the different dimensions of sustainable development in the medium–long term. In Europe, environmental action has been defined as primary and urgent and the activities connected to this action have not stopped even in the peak of the pandemic. In fact, in the first quarter of the year 2020 the milestones of the European Green Deal were presented, including among the others: the proposed *European Climate Law* (EC, 2020b), the regulation on the *EU Taxonomy for Sustainable Activities* (EC, 2020c) and the *New Circular Economy Action Plan* (EC, 2020d). The green rationale is confirmed in the *Technical guidance on the application of “do no significant harm” under Recovery and Resilience Facility Regulation*^4^ intended to assist national authorities in the preparation of the Recovery and Resilience Plans should ensure that each and every measure, reform and investment within the plan complies with the “*Do Not Significant Harm*” (DNSH) principle. The latter requires research and innovation activities should not be supporting or carrying out activities that make a significant harm to any of the six environmental objectives, within the meaning of Article 17 of EU Sustainable Finance Disclosure Regulation (SFRD), on the establishment of a framework to facilitate sustainable investment (EU Taxonomy Regulation).

The interest in green and sustainable finance is rising very fast among investors worldwide, and several voluntary private initiatives have tried to create some market standards. Policymakers have also been very active in launching numerous regulatory and non-regulatory initiatives at global or local level. To avoid market fragmentation, there is a demand for greater harmonization among the different measures. There is also a need to increase the standardization and disclosure of non-financial information published by companies and used to evaluate the risks. Doing so will help to increase data availability, to make data more comparable, and to bring more transparency and clarity to investors. Given that climate change and environmental degradation are global challenges, international cooperation is in the common interest; the European Union is actively promoting this through the International Platform on Sustainable Finance.^5^

To make the European Green Deal working effectively, EU policy-making will not be enough. A positive outcome will be related to an efficient exchange of Environmental, Social and Governance (ESG) data and information between companies, financial analysts and investors (Amel-Zadeh & Serafeim, 2018; Atkins et al., 2015; Bizoumi et al., 2019). However, current metrics often seem insufficient and companies face many difficulties while disclosing non-financial information (Cho et al., 2015; Capelle-Blancard & Petit, 2017; Boiral & Henry, 2017). With the outbreak of the COVID-19 crisis, some Social ESG metrics are resulting less representative of the reality that many companies are facing. That is why it is important to test these metrics and keep the dialogue between companies, financial analysts and investors open with the purpose to ensure that companies are using them in a dynamic and strategic way (Cheng et al., 2021). The current crisis should therefore be considered by companies, investors, financial analysts and other market players like an opportunity to change mindset and to use ESG metrics and materiality as an activity to inform corporate strategy (Broadstock et al., 2020; Cheng et al., 2021), not just reporting and use ESG disclosure for orientating capital flows toward impact investment to finance the economic recovery (Capelle-Blanchard & Petit, 2019; Tamimi & Sebastianelli, 2017).

Given these considerations the aim of this study is to assess and investigate whether the current metrics methodology is valid and will survive the COVID-19 crisis by adopting a qualitative research approach. This analysis can offer a useful insight on ESG integration in the business context by providing signals to practitioners and policymakers to make mandatory some specific ESG indicators. Particularly, the research objectives of this paper are threefold: (1) to investigate the impact of COVID-19 on ESG metrics and their effectiveness and usefulness for companies, financial analysts and investors; (2) to theorize corporate reactions in the significance of ESG metrics for businesses during and after the pandemic crisis; (3) to interpret the findings of interviews with top managers of a sample of listed European companies through a sociological lens. The motivations of this study are linked to the primary and urgent need to assess the current state of corporate reporting practices affected by the pandemic crisis by identifying and highlighting possibilities for changing some sustainability disclosure practices that could be useful for preparers, users, regulators and policymakers. As some previous studies confirmed, this unexpected and very critical situation may benefit the business context by pushing some changes on ESG issues to improve the corporate reporting transparency and to move toward more accountability based reporting practices by satisfying new information needs (Crovini et al., 2022; Hassan et al., 2021; Leoni et al., 2021; Lodhia et al., 2021; Rinaldi, 2022).

Our analysis focused on a sample of European companies and this choice is mainly motivated by the EU-wide initiatives on ESG issues and sustainable and inclusive growth (Tettamanzi et al., 2022) as it is explained previously. As this research is exploratory and grounded on practitioners’ perceptions a qualitative analysis can be considered appropriate (Corbin & Strauss, 1990; Gephart, 2004; Boiral et al., 2021) to provide a timely and detailed insight into the managers’ reaction to this new corporate disclosure scenario caused by the outbreak of Covid-19.

Our preliminary analysis can contribute to the literature in three ways. First, we can extend the current knowledge about the measurement of sustainability through ESG metrics and its effectiveness during the time of a global crisis. Second, this analysis can offer a first assessment of the primer responses to this unprecedented health emergency providing a different view of ESG reporting replying to different needs of users. Finally, this survey can give practical suggestions for changing the rationale on ESG reporting during the time of crisis.

The rest of the paper proceeds as follows. Section “Impact of COVID-19 on ESG Data and “Extra-Financial” Corporate Reports” and “The Changing Nature of Accountability and Responsibility During and After a Pandemic” describe the ESG reporting state respectively before and during/after COVID-19 and summarize previous research on this field. Section “Theoretical Framework” evokes an historical and sociological framework to support and justify our qualitative analysis and Sect. “Research Methodology” explains the research methods. Section “Findings” shows the main findings and Sect. “Discussion” discusses these results by establishing connections with existing literature and by identifying key challenges. Finally Sect. “Conclusion” draws some final remarks by proposing avenues for future research and highlighting the main implications of this research.

## Impact of COVID-19 on ESG Data and “Extra-Financial” Corporate Reports

In absence of a shared standardization of technical standards, their widespread and official disclosure, it is not easy to read and evaluate the corporate extra-financial data, known by practitioners as ESG data. Disclosure ESG data is now synonymous with a valuable proposition for the company which can thus demonstrate to convey the company's activity toward the creation of value in the medium–long term, with particular attention to the mitigation of ESG risks (Boiral et al., 2020; O’Dwyer & Unerman, 2020). To get the competitive advantages that can arise from sustainability, companies have to develop a certain awareness on the topic and translate it into a corporate vision and strategy that allow them to evaluate how sustainable issues can impact the business in the short, medium and long term (Adams, 2017; Buallay, 2019; Galbreath, 2013).

The integration of ESG data into company policies and practices is growing all over the world and now there are numerous guidelines that have been drawn up, among the most important, certainly include those of the United Nation Principles for Responsible Investment (UN PRI), United States Social Investment Forum (US SIF) and London Stock Exchange Group (LSEG) and more recently that of the Nasdaq, the Luxembourg Stock Exchange (LuxSE) and the Stock Exchange of Hong Kong Limited (HKEX). The reporting on ESG information still remains mostly on a voluntary basis, although significant steps have been taken, especially from Europe which holds a leading position in the sustainable sphere. If, on the one hand, the legislation on EU taxonomy for environmentally friendly activities is being finalized, on the other, a complete reception of the NFRD (EC, 2014) by all member countries is expected. The first European initiative will allow the definition of ESG data in terms of reliability and comparability; the second will certainly allow a greater and more widespread communication of ESG data. The EU committed to review the Non-Financial Reporting Directive in 2020, as part of the strategy to strengthen the foundations for sustainable investment. It is important to improve the framework and to harmonize between the regulations (such as EU 2019/2088, EU taxonomy for sustainable activities and European Climate Law) to ensure credibility and effectiveness. A standard is required at this stage to have ESG data consistent, comparable by sector and along the time horizon and data assurance may be required in the future, to avoid the proliferation of estimates from different external providers, different approaches that can undermine the stability of the market itself. On 21 April 2021 the European Commission published a new package of measures within the framework of the EU Action Plan for sustainable finance and climate neutrality objectives by 2050 which includes the proposal for a Directive on Corporate Sustainability Reporting (CSRD) which intends to introduce more rigorous transparency requirements on corporate sustainability, with more uniform reporting standards (EFRAG tasks), which guarantee the comparability of information for consumers, lenders and investors. Among these, it is required to communicate sustainability information with a digitally tagging in machine-readable format. Digital tagging is essential to seize the opportunities that digital technologies present to fundamentally improve the way sustainability information is used.

Among the main initiatives included in the Action Plan, the European Commission has entrusted the European Financial Reporting Advisory Group (EFRAG)^6^ with setting up a European Laboratory (Lab) whose objective is to encourage innovation and the development of best practices in corporate reporting, including environmental accounting. In this Lab, companies and investors can share best practices in sustainability reporting, such as reporting on climate in line with the recommendations of the Financial Stability Board's Task Force on Climate-related Financial Disclosure (TCFD).^7^ The European Commission issued a mandate to EFRAG for undertake preparatory work for possible EU sustainability reporting standards in a revised NFRD and on 8 March 2021, EFRAG published its final report proposing a roadmap for the development of a comprehensive set of EU sustainability reporting standards. The proposed roadmap comprises 54 detailed proposals describing the scope and structure of future sustainability reporting standards that contribute to the achievement of the EU policy objectives. The proposals do not to set out specific disclosure requirements, indicators or metrics, which is a task for EU’s future standard setter. In summary the key conclusions are the following:

- the EU has a unique sustainable development and sustainability reporting landscape which constitutes strong foundations for standard-setting. The proposals build on EU specific overarching principles and an EU specific combination of building blocks.
- Standard-setting should be built on robust EU conceptual guidelines, addressing public good alignment, expected qualitative characteristics of information, relevant time-horizons, clear boundaries, double materiality and connectivity between financial and sustainability reporting.
- The overall target architecture of standards should be coherent and comprehensive and reflect appropriate layers of reporting (sector-agnostic, sector-specific and entity-specific), relevant reporting areas and a coverage of sustainability topics classified under an ESG categorization. Presentation should preferably be organized under ‘sustainability statements’ and digitization should be considered from the start.
- The standard-setting roadmap toward the target architecture should be implemented in realistic phases. However, the first-time application of the revised Directive should benefit from a robust first set of ‘core’ standards.
- Finally, there is significant merit in promoting a mutually reinforcing cooperation between EU standard-setting efforts and international initiatives or fora.

An urgent action on the social dimension definition is necessary as COVID-19 emergency remarked. A definition of a social due diligence, that each operator must promptly follow with clear references in the standards and possibly in indicators to be reported. Moreover, supply chain due diligence with defined standards and indicators is necessary in order to have data very difficult to compute at the moment, such scope 3 GHGs. Moreover, availability of non-financial information in a digital format will be a benefit for investors and analysts (La Torre et al., 2018). Allow information be machine-readable will ensure investors and financial analysts cost saving in data processing and in reporting process, greater speed, reliability and accuracy of data handling, improved analysis, and better quality of information for the decision-makers (AIAF, 2020; Ricci et al., 2020).

At the global level, we can recognize an increasing emphasis on sustainability corporate reporting nudged by the pandemic crisis. For example, during the 37th meeting of UNCTAD’s intergovernmental working group on International Standards of Accounting and Reporting (ISAR) UNCTAD Secretary-General Mukhisa Kituyi said: “At this difficult time, the task of promoting high-quality reporting on the financial and non-financial performance of enterprises has become more important than ever.” (https://unctad.org/news/sustainability-reporting-central-achieving-global-goals-post-pandemic). Many experts confirmed that an increasing high-quality reporting on the financial and non-financial performance of companies can help boost sustainable development (Whitelock, 2019) and recovery from COVID-19. In this perspective different initiatives in various contexts can be mentioned to provide a brief summary on how corporate reporting can react to an unprecedented global crisis (see below Table 1).

**Table 1:** List of some initiatives on the impact of Covid-19 on sustainability corporate reporting

Organization	Title/reference	Notes
Value Reporting Foundation and Integrated Reporting Framework, 2020	*Integrated reporting post Covid-19, 11 May 2020* https://www.integratedreporting.org/news/integrated-reporting-post-covid-19/	Consider the value creation model of the IIRC Framework. How would your company respond differently to the different elements of that model following the Covid-19 pandemic? The reflection has to start with the impact (output and outcomes) part of the model, assessing what your company accomplished with reference to each of the different capitals
GRI, 2020	*Understanding your sustainability impacts during COVID-19, June 2020 *https://www.globalreporting.org/news/news-center/2020-06-17-understanding-your-sustainability-impacts-during-covid-19/	Webinars on role of transparency in the transition to a ‘new normal’
Integrated Reporting Committee of South Africa, (IRC), 2020	*FAQ: Reporting in a time of crisis* https://integratedreportingsa.org/ircsa/wp-content/uploads/2020/09/FAQ-Reporting-in-a-time-of-crisis-17-August-2020.pdf https://integratedreportingsa.org/integrated-reporting/reporting-in-a-time-of-crisis/	Dedicated website with examples and FAQs for supporting preparers in improving corporate reporting in times of COVID-19
KPMG, 2020	*Sustainability reporting during COVID-19 Pandemic* https://assets.kpmg/content/dam/kpmg/in/pdf/2020/05/sustainability-reporting-during-covid-19-pandemic.pdf	A new approach: Recalibrating to a post COVID-19 compatible reporting strategy. The corporate experience and response through the progression of the pandemic can be bucketed into: Respond, Relief, Recover and Resilience. This approach can anchor a company’s transition from the current corporate reporting scheme to a post COVID-19 compatible reporting scheme aligned to the shift in stakeholder requirements (p. 1)
University of West Scotland (UWS), 2020	*Integrated thinking on macro and micro levels in UK HEIs during the Covid-19 crisis—insights from the University of the West of Scotland (UWS)* https://www.advance-he.ac.uk/news-and-views/all-together	Support to communities and businesses on 'managing in crisis', 'working virtually' and 'responsible businesses' Integrated thinking at HEI
EAUC The Alliance for Sustainability Leadership in Education	*Businesses Corporate Social Responsibility (CSR) Duties Towards Communities in the Covid-19 crisis* https://www.eauc.org.uk/7017	On the basis of literature this blog offers a definition of CSR as it meets the current COVID-19. “CSR is the continuing commitment by businesses to behave fairly and responsibly and contribute to economic development whilst improving the quality of life of the workforce and their families, as well as of the local community and society at large”
United Nations Conference on Trade and Development, (UNCTAD), 2020, UNCTAD-ISAR and IAAER, 2021	*Sustainability reporting central to achieving global goals post pandemic*, 11 November 2020, blog on website https://unctad.org/news/sustainability-reporting-central-achieving-global-goals-post-pandemic https://unctad.org/system/files/official-document/diae2019d1_en.pdf https://unctad.org/meeting/unctad-isar-iaaer-workshop-impact-covid-19-company-financial-and-sustainability-reporting	Increased role of both financial and sustainability reporting in achieving the UN’s Sustainable Development Goals (SDGs) after the coronavirus pandemic. Mention on the UNCTAD Guidance for aligning sustainability reporting and SDGs (*Guidance on core indicators for entity reporting on contribution towards implementation of the Sustainable Development Goals, May 2019*)
International Federation of Accountants, IFAC, September 2021	*IFAC Calls on G20 Leaders to Focus on Sustainability Reporting and Public Sector Integrity* https://www.ifac.org/news-events/2021-09/ifac-calls-g20-leaders-focus-sustainability-reporting-and-public-sector-integrity	IFAC defined two key actions for G20 leaders to focus on as COVID-19 persists: supporting the IFRS Foundation’s initiative on sustainability standards, and championing public financial management
UN Climate Change,, October–November 2021	*COP 26 Outcomes, Transparency and Reporting, Glasgow* https://unfccc.int/process-and-meetings/the-paris-agreement/the-glasgow-climate-pact/cop26-outcomes-transparency-and-reporting	One of the key achievements of COP26 include the finalization of the "Paris Agreement rulebook". This set of rules lays out how countries are held accountable for delivering on their climate action promises and self-set targets under their Nationally Determined Contributions (NDCs)

*Source:* Authors’ elaboration. (Please note that this list is not exhaustive)

## The Changing Nature of Accountability and Responsibility During and After a Pandemic

Investors must re-prioritize engagement to focus on COVID-19 and pay more attention to the ‘S’ and the ‘G’ in the ESG acronym. Most engagement activities with affected companies and sectors should be re-focused on issues relating to COVID-19 and the response to it. Engagement on other topics (as ecosystem services, biodiversity, and species extinction) should be postponed, where possible, to allow top management and Board of Directors to focus on both, health and economic, crisis.

The EU Taxonomy, approved by the European Parliament on 18 June 2020, will be essential to pursue the engagement on these issues, which has been defined by 2020, and are part of the framework of the European Sustainable Strategy. Clear and shared KPIs need to set common goals and be effective. Finally, it is necessary to carry out researches about the gravity of the problems that undermine the livability of the earth, the existence of peoples and the ability of our society to exist. The health crisis has deeply affected our system and has brought to light many weaknesses and shortcomings that everyone has become suddenly aware and afraid of. At the beginning of the pandemic, it does not seem that biodiversity was considered as an economic priority, as well the apparent loss of importance of the climate crisis.

In the short term, also the institutions called to greening the economy operate in areas that require immediate intervention to restore normal activities and the life that every human being has currently lost in the lockdown situation. The longer-term vision and planning/strategy must necessarily be considered to define a re-start that also include climate change and biodiversity among the core elements. Currently, however, immediate priorities must considered to save human lives and providing economic and financial relief to support the most vulnerable subjects, therefore COVID-19 has affected investors demand mainly related following topics such as:

1. how companies support their employees and customers during this crisis;
2. what sacrifices have done to ensure full salaries to the workforce;
3. managers postpone or decline their own salary packages so that workforce can be partially paid;
4. what companies have done to help small business companies decided to pay small businesses quicker and bigger companies slower;
5. Companies relax payment terms for their customers to help them to conserve cash.

How a company responds could increase their reputation, investors’ confidence and make the company more resilient to the market shock. Obviously, not all answers are relevant within the context of COVID-19, employment and supply chain practices are expected to be core areas. In addition, modalities to repositioning their operations and products will be a relevant consideration. Avoiding lay-offs, providing flexible work schedule, and paid sick leave could allow the companies to be more resilient in the adversity as they might be able to maintain high employee productivity while mitigating costs by avoiding employee churn.

Similarly, those companies with well managed logistics department, efficient procurement into their supply chain, might be able to respond more quickly adapting their supply chain to avoid costly production halts. Finally, companies that change their research and development (R&D) or production efforts to create vaccines or shift manufacturing to produce test kits, ventilators, sanitizer, or other health crisis-specific services or products might signal an ability to be responsive and innovative thereby finding new ways to satisfy emerging demands. Particularly some industries such as automotive, textiles, fashion, chemical and manufacturing have been able to partially or completely reconvert their production during the pandemic crisis. The change of business activities for providing needed products and services can improve the degree of customer focus by enhancing brand loyalty and customer satisfaction. For the above reasons, firms that exhibit more positive sentiment around their human capital, supply chain and operational crisis response might earn investor confidence.

These impacts are stronger for companies that receive more coverage from external news (media, blogs, industry publications, financial analysts, etc.) consistent with more attention on these responses being associated with more significant reaction from the financial markets.

The large impact of Covid-19 on businesses can be interpreted as a significant driver for stimulating companies during pandemic to implement Corporate Social Responsibility in its real meaning by contributing to society (García-Sánchez & García-Sánchez, 2020). The Covid-19 crisis offers a great opportunity for observing differential corporate responses (Schaltegger, 2021) and for highlighting companies that are making significant and credible commitments to their stakeholders’ relationships by signaling resilience to investors (Cheema-Fox et al., 2021).

In this perspective, some recent studies demonstrated some positive externalities of pandemic crisis in the business context, such as human management practices that can re-engage employees in CSR (Aguinis et al., 2020), or by reconfiguring the role of business in society (Brammer et al., 2020) or by positively affecting strategic marketing approach (He & Harris, 2020). The pervasive consequences of Covid-19 crisis in society are generating businesses that more oriented to authentic and credible CSR practices and a real commitment to social problems (García-Sánchez & García-Sánchez, 2020).

## Theoretical Framework

The effects of a global crisis on businesses can boost different companies’ reactions and changes in accounting and accountability, as prior literature demonstrated (Baker, 2014; Dillard & Vinnari, 2017; Sinkovics et al., 2015). From an historical perspective we can found a similar understanding of the current pandemic in *De Rerum Natura* (first century BC), one of the most famous Lucretius’s work. The description of the Athens plague that struck Attica in 430 BC. allows the poet to offer a view of nature hostile to man which recalls the materialistic conception of the Greek philosopher Epicurus by arguing the question of “culpa naturae” or the wickedness of nature toward man (*Lucretius, V, 195–199*). Similarly, the current pandemic crisis brings out the negative effects of nature on man but which would have resulted from man's destructive action on the environment. Like most of the emerging diseases such as Ebola, AIDS and SARS, COVID-19 hopelessly signals the critical relationship between pandemics and the “boomerang” effects of the ecosystems destruction (WWF Italy, 2020). The man with his activities has significantly altered three quarters of the emerged lands and two thirds of the oceans, changing the planet to determine the birth of a new era called “Anthropocene” (Bebbington et al., 2020). The huge impact of the pandemic crisis on planet and human life is increasingly highlighting the importance to quantify and evaluate as much as possible rationally the current and future consequences of systemic shocks. By adopting a *materialistic philosophical approach*, all countries and organizations have to develop and adapt measurement systems to manage this uncertainty. As an interesting sub-field in sociology argued, the process of “quantification”, i.e., the production and communication of numbers, can produce many social implications (Espeland & Stevens, 2008), particularly in times of crisis (Lai et al., 2014; Sinkovics et al., 2016). By conceptualizing the quantification as a social action it is possible to analyze the “doing of numbers” and the different aims and meanings in multiple contexts (Espeland & Stevens, 2008; Mennicken & Espeland, 2019). Given the enormous proliferation of social measures that can evaluate performance of individuals and organizations, measures in firms can make them accountable and auditable by the increasing use of quantitative indicators (Hoskins, 1996; Power, 1994, 1997; Strathern, 1996). As the use of indicators can benefit in different ways, such as making relevant information more accessible for multiple users, stimulating organizations to improve by providing important feedback on policies, it is crucial to focus on the potential changes to face and manage uncertainty. Given that measures have to continually monitor and interpret different phenomenon they can elicit reactions from users that are involved in the objects they measure (Espeland & Sauder, 2007). By using the sociological framework by Espeland and Sauder (2007) we identified two different patterns, *reactivity* and *reflexivity* in order to evaluate and interpret our findings in the light of *intended* and *unintended* consequences of the pandemic crisis. The increasing production of quantitative measures during a systemic shock can produce harmful implications, such as alterations in status system, work relations, production of inequalities (Espeland & Sauder, 2007). According to Espeland and Sauder (2007) we decided to analyze the reactivity of measures by identifying the mechanisms of this reactivity through a qualitative analysis of some anonymous semi-structured interviews. In this way we can capture how indicators react to the pandemic and provide a generalized account of the situation and how respondents interpret indicators for defining objectives and justifying their decisions. By adapting the sociological framework suggested by Espeland and Sauder (2007) we analyzed the mechanisms of reactivity by evaluating two patterns, i.e., *self-fulfilling prophecy* and *commensuration*. The first one (Merton, 1968) can be defined as “processes by which reactions to social measures confirm the expectations or predictions that are embedded in measures or which increase the validity of the measure by encouraging behavior that conforms to it” (Espeland & Sauder, 2007, p. 11). *Commensuration* is the second mechanism by which qualities are converting in quantities “that share a metric, a process that is fundamental for measurement (Espeland & Sauder, 2007, p. 16). These mechanisms can help to understand how measurement systems interact with the real objects they measure (Clementino & Perkins, 2020) and react to the COVID-19 crisis.

## Research Methodology

As stated in the introduction, sometimes metrics are unable to present a complete picture of company’s practices and performance on ESG. At the same time, many recent studies demonstrated that non-financial/sustainability reporting is very weak, especially in some areas such as risk management and board oversight (Econsense, 2018; Alliance for corporate transparency, 2019; TCFD, 2019; EUROPEAN LAB@EFRAG, 2020). To achieve better outcomes, it is important to keep a continuous dialogue between investors, financial mediators, and companies on how to best mirror company’s practices in a defined framework or metrics Amel-Zadeh & Serafeim, 2018). Especially, in the current context of the COVID-19 outbreak a dynamic approach to it is key to understand which metrics will survive the current pandemic crisis and are more relevant in the current situation where companies are operating.

To test these assumptions, we have conducted a number of anonymous interviews with companies, i.e., heads or managers of sustainability/CSR departments, to understand from practice how ESG integration is being undertaken by companies and how did Covid-19 impact it. We selected companies that are part of the CSR Europe network (CSR Europe, 2021).^8^ Our focus is on the European companies as the European Union context is particularly involved in several projects, actions and policies on sustainability and ESG issues mentioned in the previous sections. The selection was based on companies representing the sectors that may be considered high environmental sensitive (see Table 2).

**Table 2:** Sectoral composition of the sample

Company #	Company industry	Interviewee’s profile	Code
1	Energy	Head of Sustainability Planning and Performance Management	R1A
		Head of Sustainability Stakeholder engagement	R1B
		Head of Sustainability Reporting	R1C
2	Energy	Head of Sustainability	R2A
		Head of Environment	R2B
3	Cement	Sustainability Integration Manager	R3A
		CSR Director	R3B
		Sustainability Performance Analyst	R3C
4	Cement	Head of Sustainability	R4A
		Quality Assurance and Sustainability Director	R4B
5	Chemical	Deputy Chief Sustainability Officer	R5A
		CSR manager	R5B
6	Oil & Gas	Head of CSR Reporting within the Investor Relations department	R6A
		CSR manager	R6B

*Source*: Authors’ elaboration

The research method involved three steps. Firstly, we sent the questionnaire to selected companies by email and then we conducted a series of interviewees with senior level managers for discussing and asking them for more details about the responses we collected in the first phase. Given restrictions and limitations of the pandemic crisis, we conducted the interviews via whichever media platform was most readily available to the interviewees. Therefore, all interview data was gathered by on line meetings through Microsoft Teams and Skype. All meetings lasted at least 1 h. Additionally these interviews were enriched by email dialogue in order to clarify some points of discussion. A total of 14 managers that are senior practitioners in leading roles and are actively involved in ESG issues in different ways are interviewed. We assigned codes to each manager that are displayed in Table 2.

The interviews were structured around the questionnaire created from the WEF and NASDAQ’s ESG Reporting Guide (Nasdaq, 2019,9), expanded with more forward-looking and situational questions (see “Appendix 1”). In our exercise, we considered mostly metrics related to Environmental issues, but, to make our analysis more complete, and updated in relation to the current situation we decided to include as well one metric under Social pillar and one under Governance. In particular, the questionnaire (“Appendix 1”) focused on six clusters: (1) Emissions, (2) Energy, (3) Water and Environment Management, (4) Board and Management Oversight (5) ESG Reporting, (6) Health and Safety and we decided to assign to each cluster the ESG dimension: the first, the second and the third (Environmental), the fourth and fifth (Governance) and finally the sixth (Social). In the third phase, during the interviews, the companies were also asked to go beyond the questionnaire and indicated their perspectives and comments on the future of ESG metrics and in general on sustainability reporting.

The methodology seems a good first step to give a clear picture on where its metrics are enough clear and where there is still work to do in the definition of well-defined metrics on ESG practices.

## Findings

As an overall result, we found that all companies have demonstrated a high level of maturity in reporting ESG information, and in particular in disclosing their activities related to the six clusters that have been selected for this analysis (see “Appendix 1”).

Generally speaking, the interviewees noticed that sustainability and corporate social responsibility have currently reached sincere attention of top management and public opinion and this increased largely the quality of ESG reporting. “COVID-19 has increased this attention and next reporting exercise will have to present even better what is truly **material** for the company and how **resilience** is the business to any type of ESG-related crisis that—as it happened for the COVID-19 outbreak—can have strong **financial and non-financial** impacts for the company”. (R1C—Cluster 5) (Emphasis added).

It is notable that “materiality” and “resilience” represents the main drivers of the interviewees’ responses. By adopting the institutionalism theory, the first one can be linked to the normative pressure and the second one, i.e., the resilience, to institutionalism diversity and polycentricity (Aligica, 2013). Transferability as well as operationalization and implementation of resilience propositions have to be developed for increasing the intercultural transferability of resilience thinking into organizational practices (Garschagen, 2013).

The analysis of the interviewees’ revels an initial assessment of the main remarks shown by companies in some of the clusters as reported below.

### COVID-19 Highlighted the Importance of Indicators on GHG Emissions and the Urgent Need to Improve Related Indicators

All respondents argued that the companies have a mature approach in disclosing information about their GHG emissions with some differences. The health emergency has highlighted some weaknesses, for instance, not all the companies in the past disclosed info about Scope 1, 2 and 3 emissions—they will start in the next reporting years. Improvements in GHG emissions are monitored over term and audited. In some cases, e.g., cement and energy industries, they are compared to sector average but not always, as in the case of the chemicals. In this case the lack of comparison is due to the fact that no international sector average is available. The long debated issue about the comparison of ESG indicators at a global level has been exacerbated by the current crisis as a manager critically argued: “Despite a strong support to Paris Agreement objectives and Sustainable Development Goals, I underline that being each of these two frameworks directed to **countries**, you cannot speak of company alignment to them”. (R5A—Cluster 1) (Emphasis added). To emphasize the need of standardizing ESG information all the companies align their disclosure to the TCFD Recommendations (often integrated to NFRD). It is worth mentioning that most of the companies incorporate climate-related risks into overall risk management. In the same perspective some of the companies are starting their preparation to comply with the new EU Taxonomy Directive.

### COVID-19 Accelerated the Link Between Climate Change and Social Issues in the ESG Indicators Framework

COVID-19 emphasized the interconnectedness between climate-related indicators and human rights or social-related metrics, as most of managers stated. “We underline the necessity of disclosing information and indicators about the impact of climate change policies to human rights and other social issues”. (R2B, R6A, R6B—Clusters 1, 2, 3 and Cluster 6). The same should be done for other environmental objectives to consider transition measures and ensure both inclusive and just transition.

### COVID-19 Assigns a High Priority on Environmental Management in Terms of Both Risk and Opportunity

All the companies set business targets for environmental management, also at global level. We can notice that environmental management is scoring high in the priorities of some companies that monitor it over with quantitative data. Regarding water management, not all the companies consider it a material issue. Nevertheless it is worthwhile to note that environmental management indicators often are not considered enough to disclose information about how the company address biodiversity-related issues (R1A, R4A—Clusters 1, 2, 3 and Cluster 4).

A pervasiveness of environmental management in all business functions is revealed by most of managers. “Environmental risks and their potential financial impacts are evaluated along all step of the business activity through methodologies like the Sustainable Portfolio Management which does a risk and opportunity analysis for each product in each market, including upstream and downstream activities”. (R3B, R4B—Clusters 1, 2, 3 and Cluster 4).

### COVID-19 Affects ESG Reporting Practices in Improving Disclosure on the Health and Economic Crisis

More than half of the interviewed companies is publishing an integrated report, updated every year. There are many people involved in the drafting of the document, in general by the collaboration of different teams such as Sustainability, Investors Relations, Communications, Risk Management team, and Legal team, with involvement of technical colleagues. Final approval in many cases is given by CFO (R5A—Cluster 5).

We are going to add in the next report ACTIONS related to COVID-19 but NOT new metrics or indicators so far. Discussion are starting in the summer, but how this is going to affect metrics is not clear so far”. (R1A, R2A—Cluster 5). “To report actions on how companies have transformed their business models and core activities to react to the pandemic crisis as well as support the communities (e.g. shift in production, services to support customers, etc.) companies are not waiting to do so in the annual report but do so often through their website or newsletter on a weekly or monthly basis”. (R4A—Cluster 5) “To better reflect the growing importance of sustainability and the growing commitment of a company, we suggest the inclusion in the report of metrics to assess presence of trainings on sustainability for managers and top management. This will be to showcase how much you invest in sustainability also for the future. (R1C—Clusters 4 and 5).

It is notable that the managers’ reactions are focused on a radical change in reporting practices, related to kind of report, timeliness and key sustainability indicators.

#### COVID-19 Signals the Urgent Need to Re-consider Health and Safety Indicators in the Light of Pandemics and Disasters

All companies agrees that health and safety became quickly one of the top priorities at beginning of 2020. The companies faced the COVID-19 pandemic putting in place different processes. For some sectors, like the chemicals and oil and gas, there was not a single process to identify areas of the production that are most risky to the health of the workforce in relation to COVID-19. Mostly, a facility by facility and unit by unit assessment was put in place to understand the risk.

A manager from the chemicals argued that “Guidance on hazards and risks related to health and safety and specifically on COVID-19 are issued generally at global level for the company, but it is adapted to the local situation according to the health and economic situation of the country the site is in” (R5B—Cluster 6). The strong company health and safety culture helped the employees the right tools to face a state of emergency, increasing their resilience.

Hours of training seems to be not an appropriate and reliable metric anymore because trainings are switching to online training and the time you take do undertake it cannot be measured, each person can take it at the speed they want (R1A, R1C—Cluster 6).

#### COVID-19 Impacts on Digitalization Policies During and After Pandemic Crisis

Many companies increased the share of people working from home drastically. Half of the companies had already put in place a strong digitalization policy to facilitate homeworking before the pandemic. “Homeworking policy during the pandemic was set at global level, preventing the scaling up of the pandemic in some facilities in areas at risks” (R1A, R2A—Cluster 6). Extra support for employees, including, financial support, health coverage (also for mental health) has appeared as a trend among the companies interviewed. An interesting novelty among different comments, as reported below. “Extra measures to secure health and safety of suppliers in relation to COVID depend on the type of contractors but we will apply to the ones entering in contact with the company but not in terms of giving them instructions on how they will do on their own sites”. (R1A, R2A, R5B—Cluster 6). Given that there is an urgent claim for a revolutionary re-design of the health and safety indicators system not only for employee but for all actors of the supply chain.

## Discussion

Conducted anonymously, the interviews for this study show the increasing importance of the validity of measurement in the ESG context (Semenova & Hassel, 2015) as a response to COVID-19, from both corporate board and public opinion. Companies feel that in their next reporting exercise there will be the need to address how they managed the double crises we are facing: the health and the economic ones. As well as the rebound for recovery. This will leave a lasting impression of what their true sustainability priorities are (Tables 3 and 4).

**Table 3:** Findings, clusters, previous studied and theories

Finding	Cluster	Literature	Theory
GHG emissions metrics	1	Liesen et al. (2015), Depoers et al. (2016), Comyns (2018), Chithambo et al. (2020), Ryan and Tiller (2022)	Institutional theory Legitimacy theory Stakeholder theory
Environmental management metrics	1, 2, 3 and 4	Harms et al. (2013), Muhammad et al. (2016), Dobler et al. (2015), Haque and Ntim (2018), Silvestre et al. (2018), Yoo et al. (2021), Arvidsson and Dumay (2022)	Stakeholder theory Socio-political theories
Interconnectedness between social and environmental metrics	1, 2, 3 and 6	Bui and de Villiers, 2021), Adams and Abhayawansa (2022), Larrinaga and Garcia-Torea (2022)	Institutional theory
Emphasis on materiality and resilience	5	Cheema-Fox et al. (2021), Kober and Thambar (2021), Chhatwani et al. (2022), Adams and Abhayawansa (2022)	Stakeholder theory (materiality) Broaden & Build (B&B) theory (resilience) Organizational resilience
No new metrics, not additional information in annual report	4 and 5	Zharfpeykan and Ng (2021), Severo et al. (2021), Adams and Abhayawansa (2022)	Impression management Legitimacy theory
Health and safety metrics	6	Parker (2020), Zhang et al. (2021), Ferrannini et al. (2021), Adams and Abhayawansa (2022), Saura et al. (2022	Industrial policy and sustainable human development Organizational change
Digitalization policies	6	Brenner and Hartl (2021), Ivanov and Dolgui (2021), Gupta and Singh (2021), Lichtenthaler (2021), Low and Bu (2022), Saura et al. (2022)	Social representation theory Industrial policy and sustainable human development Organizational change

*Source*: Authors’ elaboration

**Table 4:** ESG metrics during COVID-19 and managers’ reactivity

Main trends	Typology of reactivity	CSR institutional factors
GHG emissions metrics	Passive conformity	Government regulations
Environmental management metrics	Active conformity	Government regulations; Private self-regulations, associate behavior
Interconnectedness between social and environmental metrics	Active conformity	Private self-regulations; independent watchdogs
Emphasis on materiality and resilience	Active conformity	Normative expectations
No new metrics, not additional information in annual report	Active resistance	Private self-regulations, normative expectations, associate behavior
Health and safety metrics	Active conformity	Private self-regulations, normative expectations

*Source:* Our adaptation by Espeland and Sauder (2007), Di Maggio and Powell (1983), Campbell (2018), Clementino and Perkins (2020)

In more details we categorize our main interview data in the six clusters of the questionnaire (“Appendix 1”) and we can identify some connections with the previous recent literature and some selected theories.

Given these evidences, the main overall trends of this survey can be interpreted through the lens of the sociological framework by Espeland and Sauder (2007) by assessing the mechanisms of reactivity by companies on the effectiveness of ESG measures in times of COVID-19. In the table below we identified four typologies of reactivity (Clementino & Perkins, 2020) and the main Corporate Social Responsibility (CSR) institutional factors (DiMaggio and Powell, 1983; Campbell, 2018) that affect managers’ reactivity by integrating institutional theory in this theoretical analysis. The four typologies of reactivity (Clementino & Perkins, 2020; Espeland & Sauder, 2007) include different degrees of conformity, i.e., from passive to active, and of resistance, i.e., from passive to active. In the case of “conformity” the mechanism of reactivity requires to conform to government regulations (passive) or government and private self-regulations (such as internal organizational changes, implementing new policies, setting incentives, etc.). Otherwise in the case of “resistance” the mechanism of reactivity refers to a divergence between companies’ information needs and private self-regulations and normative expectations (active resistance).

This relatively new interconnectedness between businesses and quality of life is determining a radical change in the corporate sustainability boundaries to make accessible how companies are contributing to human life and safety (Parker, 2020). An extensive re-formulation of the ESG metrics is required in the light of times of crisis, as accountability and transparency linked to quantitative measures can play a critical role in the financial system (Espeland & Vannebo, 2007). Particularly, this questionnaire survey shows a strong need for better and more standardized metrics on many aspects of sustainability: biodiversity, social aspects, including diversity and looking more at the resource management and at its impact on the quality of life. Companies will, however, include new content in relation to how they managed the pandemic crisis and the rebound. Moreover the corporate response to COVID-19 needs to better assess the *interconnectivity* among the different aspect of sustainability (e.g., what is the impact of biodiversity loss and climate change on social aspects?) (Adams & Abhayawansa, 2022; Biehl & Thomson, 2020). Only in this way we will be able to ensure a just and inclusive transition, as the Sustainable Development Goals and Agenda 2030 have already pointed out (Bebbington & Unerman, 2018; Consolandi et al., 2020; Lokuwaduge & Heenetigala, 2017). Additionally, many have noticed that they are not sure if there will be the need for metrics related to resilience to pandemic. It would be better to use “horizontal” indicators than specific ones. Some companies questioned the importance of resilience over flexibility to react. While resilience means reacting to something to avoid its negative impacts, flexibility includes managing the crisis actively. Investing on a flexible model before (e.g., digitalization and teleworking policies) can save its stability of the company in times of crisis. This preliminary assessment can provide several practical implications for managers and policymakers to stimulate a radical improvement of ESG metrics enhancing some indicators rather than other ones, in a more extensive approach. Hence, the strict distinction between “E”, “S” and “G” issues should be abandoned claiming a different holistic re-design of sustainability measures by considering the relevance of the Social dimension, particularly the strong connection between safety conditions at the workplace and personal life (Atkins et al., 2020; Parker, 2020).

Moreover, an overall forward-looking reaction to COVID-19 in terms of next reporting exercises will also deal with a new debate on supply chain management (Sharma et al., 2020) and what is the responsibility of companies also over the current due diligence frameworks. ESG metrics should be re-designed along the supply chain in an inclusive and holistic approach as the European Commission announced as a new regulatory standard or framework (EC, 2020e) that has been published in 2022, i.e., EU Due Diligence Act.

## Conclusion

This research offers the first initial assessment of managers’ reaction to Covid-19 impact on the current ESG metrics framework by conducting a series of semi-structured interviews with senior practitioners of some high environmental sensitive large companies in the European context. By highlighting six clusters within 30 ESG metrics our interview data identifies different managers’ reactions to some metrics/issues affected by pandemic crisis.

These findings can provide useful suggestions for regulators, policymakers, preparers, users and managers on the main metrics to be carefully monitored during times of crisis. Additionally, this analysis has practical implications for the financial sector as the current crisis requires a systemic resolution approach. One example of this approach is the use of public finance for sustainable development, like in the case of the vigorous monetary and fiscal policies just implemented in the Eurozone which are in line with the demand to overcome the current crisis. However, this request must incorporate as well long-term strategic policies for an effective transition to a climate-resilient low-carbon economy. This is the only truly effective “vaccine” to address not only climate risks, but future social and economic risks as well.

Nevertheless this study shows some limitations. Our sample is drawn from a subjective selection on the basis of the environmental relevance of some industries and it is not representative of the overall population of companies. The data collected are affected by managers’ perceptions and are not necessarily shared by other stakeholders (Boiral et al., 2021). The number of the interviewees is relatively low but we believe that, at this phase of Covid-19, our empirical evidence can address the need for identifying the emerging corporate disclosure practices and understanding how the institutional environments can influence these ESG metrics (Rinaldi, 2022).

This research opens to further avenues for exploring managers’ perceptions on more typologies of ESG metrics, related, for example, to GRI or SASB standards. Future research could investigate the perceptions of different kinds of stakeholders on ESG metrics by carrying out larger-scale quantitative studies, on both high and low environmental sensitive sectors. More studies are needed after the Covid-19 emergency period to evaluate changes in managers’ reactions to the validity of ESG metrics.

## Appendix 1

### Questionnaire on ESG Metrics

(adaptation from WFE ESG Guidance and Metrics^10^ and ESG Reporting Guide of NASDAQ^11^).

### Cluster 1: Emissions (GHG & Intensity)

1. Is there a business target…
       ….. on scope 1, 2 and 3?
       ….. on emission intensity?
2. Is this target aligned with Paris Agreement?
3. Is this improvement monitored over term?
4. Does the company disclose information on emission rate vis-à-vis sector average?
5. Is the business targets translated to individual KPIs?
6. Is there a corporate strategy for achieving the target? (TCFD Climate Scenario)?
7. Do you use (or plan to use) the EU taxonomy on climate?
8. Is there a stakeholder engagement policy?
9. Does the company provide information on Climate-related Risks and opportunities, in line with TCFD? Are these risks incorporated in overall risk management?

### Cluster 2: Energy (Usage, Intensity, Mix)

1. Is there a business target on energy usage, intensity and mix?
2. Is the target aligned with the Paris Agreement as well as with the SDGS framework (climate and clean energy related SDGs?
3. Are these topics integrated in the risk management? Is there any related climate scenario analysis?
4. Is energy usage aligned with EMAS?

### Cluster 3: Water and Environment

1. Does the company have a business target related to water management and/or environmental operations? Is it in line with the SDGs?
2. Is there a corporate strategy to achieve it?
3. Are these targets monitored over time?
4. A water and environment sensitivity analysis for the company is available?
5. Are in place resilience policies on water management and environmental management to address the transition and physical risks may affect demand for products and services?
6. Does the company aware and respectful of local communities?
7. Are environmental risks and their potential financial impacts evaluated along all step of the business activity?

### Cluster 4: Board and Management Oversight

#### Board

1. Do the board and/or board committees consider when reviewing and guiding strategy, major plans of action, risk management policies, annual budgets, and business plans….
       ….. Climate-related issues?
       …… Energy?
       …… Environment?
       …… Water Management?
2. Do the board and/or board committees consider when setting the organization’s performance objectives, monitoring implementation and performance, and overseeing major capital expenditures, acquisitions, and divestitures…
       ….. Climate-related issues?
       …… Energy?
       …… Environment?
       …… Water Management?

#### Management

(3) How the Senior Management Teams identify environmental/climate-related issues and their impacts, risks and opportunities and implement a due diligence process?
(4) How the Senior Management Teams review the effectiveness of risk management processes for e identify environmental/climate-related issues?
(5) How often the Senior Management Teams review identify environmental/climate-related issues and their impacts, risks, and opportunities?

### Cluster 5: Governance—ESG Reporting

- Is there an ESG report and how long is it updated? Who writes the report?
- Is there a stakeholder engagement policy?
- Are there any processes for ensuring reliability of information and transparency?
- Are you planning to include in your next report new metrics related to the COVID-19 outbreak?
  E.g., actions to support your suppliers (delayed payments, shift in delivery timeline, others)
- Are you going to include in your next report if and how you transformed your business models and core activities to react to the pandemic crisis as well as support the communities (e.g., shift in production, services to support customers, etc.)

### Cluster 6: Health and Safety

- Are there any safety standards within the company?
- Has the company identified some areas of the production process that are most risky to the health of the workforce, especially in relation to COVID-19?
- Is there a process description manual used to investigate any hazards and risks related to COVID-19?
- How many hours of training are provided on safety and health? Are there any constant updates? And is an updated related to COVID-19 planned?
- Will the company describe homeworking policy effectiveness—e.g., % people working from home?
- Has the company put in place procedures (and is planning to report on them) to ensure a safe working place for the employees who need to be onsite? (e.g., measuring temperature at entrance, controlling human distancing at workplace)
- Is the company providing guidance, support and resources to work from home when possible/reduce exposure risk?
- Is the company expanding in certain cases medical cover for the covid-19 infections and support for employees mental health?
- Have you put in place measures in relation to the health and safety of your suppliers?

## Footnotes

1 The 2019 Lancet Countdown Report associates climate change with an increased spread of infectious diseases, some of which are already known as dengue and malaria. Health is aggravated by impacts due to poor food safety, extreme weather events and air pollution. For more details see https://www.thelancet.com/journals/lancet/article/PIIS0140-6736(19)32596-6/fulltext

2 The Intergovernmental Group of Experts on Climate Change in the various special reports of 2019 reiterated once again how the anthropogenic activities that cause emissions, can themselves cause direct damage to our health.

3 Health systems around the world are poorly prepared for significant outbreaks of other emerging infectious diseases, such as SARS, Zika and MERS whose rapid and massive spread aggravated by resistance to antibiotics, antivirals and other treatments leads to widespread fatalities and economic disruption.

4 https://ec.europa.eu/info/sites/default/files/c2021_1054_en.pdf. Last Accessed May 24, 2021.

5 At the global level The United States Securities and Exchange Commission (SEC) established a Task Force focused on climate and ESG issues whose initial goal is to identify any material gaps or inaccuracies in issuers’ disclosure of climate risks under existing regulations. The SEC Task Force will also analyze disclosure and compliance issues related to the ESG strategies of investment advisors and funds and coordinate the effective use of the division’s resources, including through the use of sophisticated data analytics to extract and evaluate the disclosed information in order to identify potential breaches. SEC Announces Enforcement Task Force Focused on Climate and ESG Issues, 04/03/2021, https://www.sec.gov/news/press-release/2021-42.

6 EFRAG is a private association, founded in 2001 with the encouragement of the European Commission to serve the public interest, involving national organizations that have knowledge and interest in the development of IFRS and how they contribute to the efficiency of the capital markets.

7 To help identify the information necessary for investors, lenders and insurance underwriters to adequately assess climate-related risks and opportunities, the Financial Stability Board set up a Task Force in 2015 which was asked to develop voluntary and consistent financial information on the climate useful for investors, lenders and insurers to understand material risks.

8 CSR Europe is the European Business Network for Corporate Sustainability and responsibility. CSR Europe corporate members includes 30 multinational companies across sectors and regions and 37 national partner organizations. This network reaches out to more than 10,000 companies in Europe. For more details see https://www.csreurope.org/.

9 This Guide includes 30 material ESG metrics that are categorized in the three dimensions, Environmental (E1-E10), Social (S1-S10) and Governance (G1-G10). For more details see https://www.nasdaq.com/docs/2019/11/26/2019-ESG-Reporting-Guide.pdf.
